# Native‐tissue prolapse repair: Efficacy and adverse effects of uterosacral ligaments suspension at 10‐year follow up

**DOI:** 10.1002/ijgo.14096

**Published:** 2022-02-08

**Authors:** Alice Cola, Giuseppe Marino, Rodolfo Milani, Marta Barba, Silvia Volontè, Federico Spelzini, Stefano Manodoro, Matteo Frigerio

**Affiliations:** ^1^ University of Milano‐Bicocca Monza Italy; ^2^ ASST Monza, San Gerardo Hospital Monza Italy; ^3^ AUSL Romagna, Infermi Hospital Rimini Italy; ^4^ ASST Santi Paolo e Carlo, Ospedale San Paolo Milan Italy

**Keywords:** complications, functional outcomes, long‐term follow up, pelvic organ prolapse, quality of life, uterosacral ligaments suspension

## Abstract

**Objective:**

To evaluate the 10‐year outcomes of high uterosacral ligaments suspension as a primary repair for apical prolapse and to evaluate the long‐term impact of prognostic factors.

**Methods:**

A retrospective study analyzed 10‐year follow up after repair of primary apical prolapse through high uterosacral ligament suspension. Bulging symptoms and postoperative prolapse stage II or above were considered subjective and objective recurrences, respectively. Patient Global Impression of Improvement score was used to evaluate subjective satisfaction after surgery.

**Results:**

A total of 287 women were analyzed. Ten‐year recurrence rates were 19.1% for objective recurrence and 6.3% for subjective recurrence; surgical retreatment rate was 2.1%. Premenopausal status was related to 15‐fold increased risk of developing either objective or subjective recurrence. Conversely, anterior and posterior repair were protective factors against reoperation.

**Conclusion:**

High uterosacral ligaments suspension is a safe and long‐lasting effective procedure for the treatment of uterovaginal prolapse even 10 years after index surgery. Premenopausal status and lack of anterior and posterior repair represented long‐term risk factors for surgical failure.

## 1 INTRODUCTION

Pelvic organ prolapse (POP) is the descent of the uterus, bladder, rectum, and/or bowel through the vagina. It represents a worldwide public health issue and one of the main findings in patients seeking care in pelvic floor clinics. Associated symptoms involve alteration in bladder, bowel, and sexual functions.^1^ POP management includes both conservative and surgical treatment according to stage, symptoms, and patient’s general health and wishes.^2^ However, surgical repair is considered the mainstay of POP treatment. Currently, there is a renewed interest in mesh‐free procedures because of lower costs and lack of graft‐related complications. The main criticism leveled by detractors is that native tissue procedures are associated with higher risk of recurrence in the long term. Proposed risk factors include age and other population characteristics, obstetric history, advanced prolapse stage, histologic findings, and supplementary surgical procedures performed (such as anterior or posterior repair).^3^, ^4^ Among native‐tissue techniques, high uterosacral ligaments (USL) suspension is considered a valid and effective procedure for central compartment repair. Moreover, it is versatile because it can be used for primary repair, POP recurrence, and uterus‐sparing surgery.^5^, ^6^, ^7^ However, as for other native‐tissue procedures, there is a lack of data about the long‐term follow up in terms of objective and subjective outcomes. This is even more relevant considering population aging and limited healthcare resources.

The present study represents an update of our previously published study on the 5‐year safety and efficacy of USL suspension for the primary treatment of uterovaginal prolapse.^8^ We aimed to evaluate the 10‐year effectiveness and functional results of high USL suspension as a technique for primary repair of apical prolapse. Moreover, we aimed to evaluate the long‐term impact of prognostic factors on outcomes.

## 2 MATERIALS AND METHODS

Between October 2008 and December 2012, patients who underwent native‐tissue repair of through vaginal hysterectomy followed by high USL suspension for POP in a single center were retrospectively analyzed. Patients living outside the administrative region or followed up outside the hospital at the office of a trusted gynecologist were not considered. Preoperative evaluation included a medical interview to assess obstetrical history. The presence of urinary, sexual, and bowel symptoms was defined according to International UroGynecology Association and International Continence Society standardization of terminology.^9^ A urogenital examination was performed and POP was staged according to the Pelvic Organ Prolapse Quantification system (POP‐Q).^10^

**FIGURE 1:**
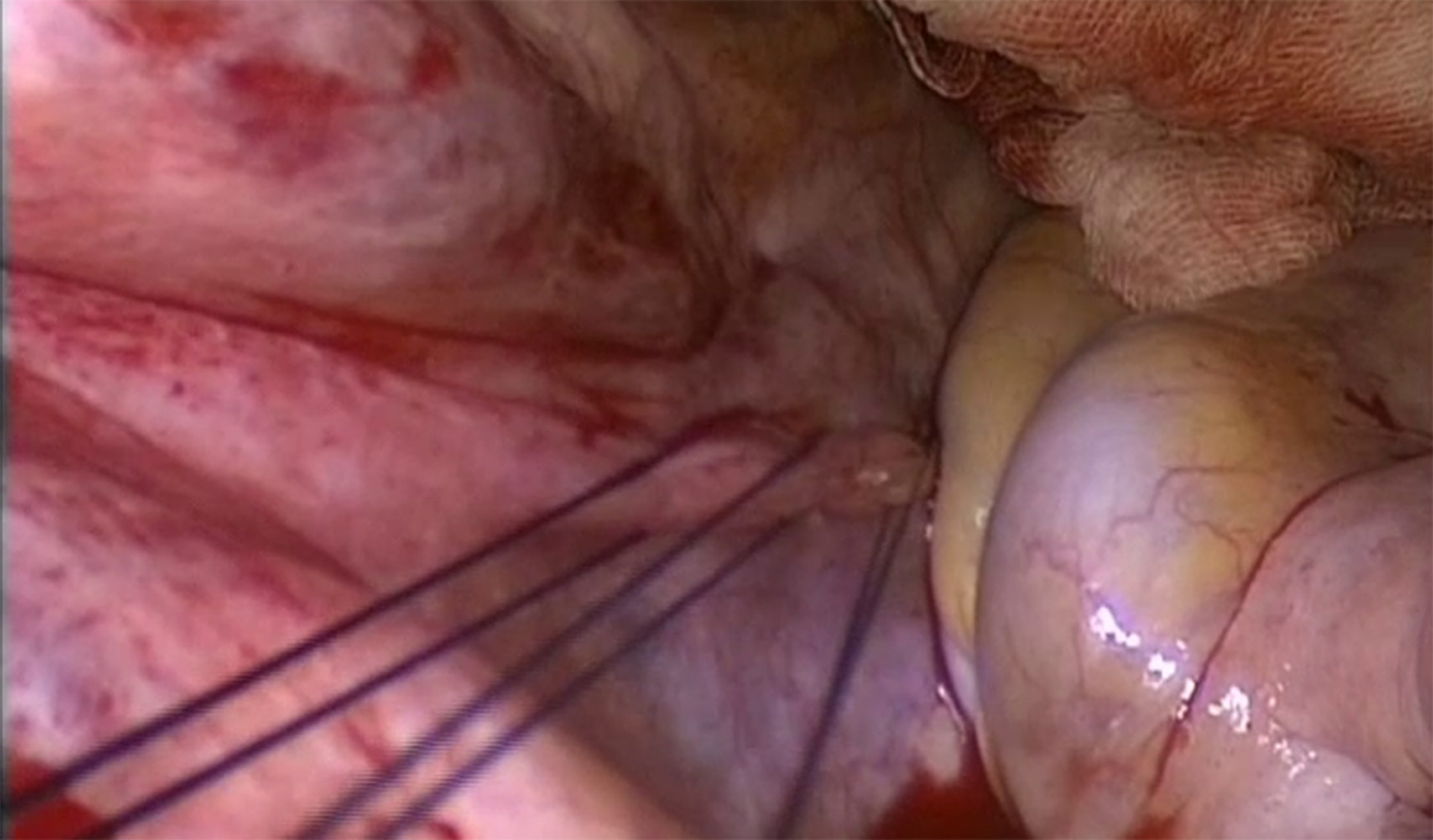
Triple transfixion of the right uterosacral ligament

Patients underwent transvaginal hysterectomy and salpingectomy; bilateral oophorectomy was performed—if technically feasible—according to menopausal status, age, oncologic risk, and patients' will. After vaginal hysterectomy, high USL suspension was performed with two or three monofilament absorbable size 0 sutures per side (Figure 1).^7^ Additional surgical procedures, such as anterior and/or posterior repair, were performed when needed. Specifically, the anterior repair was performed through midline fascia plication with non‐absorbable interrupted sutures from the level of the bladder neck to the apex of the anterior vaginal wall, and the apex of the duplicated fascia was incorporated with suspension sutures. This represented the standard procedure for POP primary repair in the case of menopausal women or premenopausal women not requiring uterine preservation during the given period. All the procedures were performed by two surgeons experienced in pelvic floor surgery. Patients were followed up 1 year after surgery and then yearly. Patients who did not perform a visit in the last year were called by telephone and scheduled for a visit. In case of refusal, we asked for the reason. Follow‐up visits included a clinical interview and a complete urogenital examination. Postoperative presence of bulging symptoms according to the specific item of the Italian version of the Prolapse Quality Of Life (P‐QoL) questionnaire (answer “A little/Moderately/A lot” to the item “Feeling a bulge/lump from or in the vagina”) was considered as a subjective recurrence [112]. A postoperative descent to stage II or below according to the POP‐Q system in any compartment or the need for reoperation was considered as objective recurrence. Patient Global Impression of Improvement (PGI‐I) score was used to evaluate subjective satisfaction after surgery.^11^ This is a seven‐point scale quality of life (QoL) questionnaire evaluating patients' satisfaction with a range of responses from 1, “very much improved” to 7, “very much worse”. QoL success was defined by both “very much improved” and “much improved” at PGI‐I score (≤2). The study was approved by the Institutional Review Board of San Gerardo Hospital in Monza, Italy (SH‐MCC 1709/2013). Data were collected from hospital‐dedicated software for patients' clinical monitoring. Data were entered into the database by one author and double‐checked by one other author. Descriptive statistics were calculated as absolute numbers with percentages for categorical variables and as median (interquartile range) for continuous ones. Differences were tested with the Student's *t* test for continuous parametric variables, with the Wilcoxon test for continuous non‐parametric variables, and with the χ^2^ test for non‐continuous variables. Evaluated factors included population characteristics (age, obesity, menopausal status), obstetric history (number of deliveries, operative delivery, macrosomic babies), preoperative prolapse stage, surgical procedures performed (anterior and/or posterior repair). Statistical analysis was performed with JMP 7.0 (SAS Institute, Cary, NC, USA). A *P* value less than 0.05 was considered significant.

## 3 RESULTS

A total of 402 records were identified. Forty‐nine women living outside the administrative region or followed up outside the hospital at the office of a trusted gynecologist were not considered. As a consequence, 353 women who underwent vaginal hysterectomy and high USL suspension in the study period were evaluated. Most women had a uterovaginal prolapse stage 3 or above according to POP‐Q. The total complication rate was 4.0%, with ureteral kinking being the most frequent complication (2.3%). This was identified by intraoperative cystoscopy in all cases and managed with ureteral stenting or intraoperative revision of the sutures. Other complications included: hemoperitoneum (0.8%); bladder perforation (0.3%); vaginal cuff abscess (0.3%); and urinary retention requiring suburethral sling cut (0.3%). Sixty‐six patients were lost at follow up (18.7%) because they were severely ill, dead from causes not related to pelvic floor surgery, or unable to be contacted. The remaining 287 patients were analyzed. The median follow‐up time was 120 months (100–130 months). Population characteristics are reported in Table 1. Surgical procedures performed are shown in Table 2. Mean operative time was 100 min (85–120 min) and blood loss was 250 ml (150–300 ml). Long‐term outcomes are reported in Table 3. Overall, anatomic recurrence at 10 years was found in 55 (19.1%) patients. The anterior compartment was the site with the highest rate of recurrence (45 patients; 15.7%), followed by the posterior (19 patients; 6.6%) and the central (7 patients; 2.5%) compartments. Symptoms of prolapse were reported by 18 (6.3%) patients, and surgical retreatment for prolapse recurrence was required by only 6 (2.1%) women. None chose/required pessary use. Comparison between preoperative and 10‐year postoperative vaginal profile according to the POP‐Q system is shown in Table 4. We noted a persistent improvement in all POP‐Q points at the 10‐year time point compared with the preoperative assessment, with the exception of total vaginal length, which was shorter after surgery (10.3 vs. 8.7 cm; *P* < 0.001). Even in the long term, native‐tissue prolapse repair did not result in any detrimental effect on functional outcomes (Table 5). Notably, a persistent positive impact was observed—besides bulging symptoms—on stress incontinence and voiding symptoms rates (*P* < 0.001). Multivariate analysis demonstrated a significant impact of considered risk factors on long‐term outcomes (Table 6). Specifically, premenopausal status involved a 15‐fold risk of developing either objective or subjective recurrence. Moreover, anterior and posterior repair at the time of prolapse surgery was protective against reoperation for prolapse (odds ratios 0.06 and 0.13, respectively).

**TABLE 1:** Population characteristics^a^

Characteristics	
Age, years	64 (57–70)
Vaginal deliveries	2 (2–3)
Maximum birth weight, g	3600 (3300–3900)
Operative delivery	41 (14.2%)
Premenopausal status	31 (10.8%)
Body mass index^b^	24.6 (22.5–26.8)
Anterior prolapse stage	2 (2–3)
Central prolapse stage	1 (1–3)
Posterior prolapse stage	1 (1–2)

^a^ Continuous data are presented as median (interquartile range); non‐continuous data are presented as absolute frequency (relative frequency).

^b^ Calculated as weight in kilograms divided by the square of height in meters.

**TABLE 2:** Surgical procedures performed^a^

Procedure	Frequency
Hysterectomy	287 (100%)
High uterosacral ligament suspension	287 (100%)
Anterior repair	256 (89.2%)
Posterior repair	214 (74.6%)

^a^ Data are presented as absolute frequency (relative frequency).

**TABLE 3:** Long‐term outcomes^a^

Outcome	Frequency
Anatomic recurrence	55 (19.2%)
Anterior recurrence	45 (15.7%)
Central recurrence	7 (2.4%)
Posterior recurrence	19 (6.6%)
Subjective recurrence	18 (6.3%)
Quality of life recurrence	17 (5.9%)
Reoperation	6 (2.1%)

^a^ Data are presented as absolute frequency (relative frequency).

**TABLE 4:** Anatomical outcomes according to POP‐Q system: Comparison between preoperative and long‐term findings^a^

Outcome	Preoperative	10‐year outcomes	*P* value
Aa	+1.0 ± 1.5	−1.9 ± 0.9	<0.001
Ba	+1.3 ± 1.6	−1.9 ± 0.9	<0.001
C	−0.2 ± 2.8	−6.8 ± 2.1	<0.001
gh	3.6 ± 0.5	3.1 ± 0.6	<0.001
pb	2.9 ± 0.4	3.0 ± 0.3	0.036
tvl	10.3 ± 1.4	8.7 ± 1.6	<0.001
Ap	−1.5 ± 1.1	−2.5 ± 0.7	<0.001
Bp	−1.5 ± 1.2	−2.5 ± 0.7	<0.001
D	−4.6 ± 2.8	—	—

Abbreviations: POP‐Q, Pelvic Organ Prolapse Quantification system.

^a^ Data are presented as mean ± standard deviation.

**TABLE 5:** Functional outcomes: Comparison between preoperative and long‐term findings

	Preoperative	10‐years outcomes	*P* value
Bulging symptoms	271 (94.4%)	18 (6.3%)	<0.001
Urge incontinence	54 (18.8%)	62 (21.6%)	0.467
Stress urinary incontinence	111 (38.7%)	52 (18.8%)	<0.001
Voiding symptoms	142 (49.5%)	56 (19.5%)	<0.001
Constipation	82 (28.6%)	69 (24.0%)	0.255
Sexual activity	166 (57.8%)	145 (50.5%)	0.094
Dyspareunia^a^	32 (19.3%)	31 (25.3%)	0.673

^a^ In sexually active patients.

**TABLE 6:** Multivariate analysis

	Anatomic recurrence	Subjective recurrence	QoL recurrence	Reoperation
Age ≤ 50 years	ns	ns	ns	ns
Premenopausal status	*P* = 0.002 OR = 15.56	*P* = 0.024 OR = 15.18	ns	ns
Obesity	ns	ns	ns	ns
Parity ≥ 4	ns	ns	ns	ns
Operative delivery	ns	ns	ns	ns
Macrosomic newborn	ns	ns	ns	ns
Preoperative POP stage ≥ 3	ns	ns	ns	ns
Anterior repair	ns	ns	ns	*P* = 0.036 OR = 0.06
Posterior repair	ns	ns	ns	*P* = 0.044 OR = 0.13

Abbreviations: ns, not significant; OR, odds ratio, POP, pelvic organ prolapse; QoL quality of life.

## 4 DISCUSSION

Long‐term outcomes of reconstructive pelvic surgery are not well known, which can be relevant when considering native‐tissue repair, in which the recurrence rate is supposed to be unsatisfactory. The present study reports the combination of subjective, objective, functional, and QoL outcomes of USL suspension for the treatment of uterovaginal prolapse at 10‐year follow up. This represents—to the best of our knowledge—the first work evaluating the outcomes of USL suspension, and of native‐tissue repair for uterovaginal prolapse, 10 years after the index surgery. We found that USL suspension is a long‐lasting, effective, and safe procedure, with beneficial impact on functional outcomes. In our population, we demonstrated satisfying subjective (93.9%) and objective (80.9%) cure rates 10 years after transvaginal USL suspension, with a minimal reoperation rate (2.1%). Patients' self‐impression of improvement was good/very good in 94.1% of women, and long‐term beneficial effects on stress urinary incontinence and voiding symptoms were observed. Multivariate analysis demonstrated that premenopausal status and the lack of anterior and posterior repair are risk factors for recurrences.

In 2011 the US Food and Drug Administration notification about the use of surgical mesh for the transvaginal repair of POP highlighted the need to re‐consider native‐tissue repair and to make an effort to report long‐term outcomes of these techniques, which are traditionally supposed to be particularly unsatisfactory despite the lack of data. Recently, good long‐term outcomes of native tissue procedures have been reported for sacrospinous ligament fixation and iliococcygeus fascia fixation.^12^, ^13^ Regarding high USL, few data for medium‐ to long‐term outcomes are available in the literature.

In our previous 5‐year follow‐up study, subjective and objective cure rates after USL suspension were 85% and 87%, respectively, with a reoperation rate of 1.1%.^14^ Similarly, satisfying outcomes were reported by other authors at follow ups ranging from 5 to 9 years. Doumouchtsis et al.^15^ reported objective and subjective outcomes 5 years after native tissue repair with USL suspension in a cohort of 42 women. Globally, 11.9% of women reported vaginal bulging. The reoperation rate was 9.5% for apical or posterior prolapse recurrence. In the remainder, 12 patients (30.7%) were found to have an anterior recurrence (at least stage 2). Silva et al.^16^ evaluated 5‐year outcomes of USL suspension for uterovaginal or vaginal vault prolapse repair in a cohort of 110 patients. After 5 years, they reported 34% loss at follow up and 84.7% anatomical success in the remaining 72 patients. Similarly, Zhang et al. ^17^retrospectively assessed the medium‐term effect of transvaginal USL suspension in a population of 79 women. After 5 years, they observed a combined objective‐subjective success rate of 86%, with 35% loss at follow up. Only two studies with a follow up longer than 5 years after USL suspension are available in the literature. Schiavi et al.^18^ retrospectively evaluated 414 patients after either modified McCall or Shull suspension. After a median of 8.9 years, they reported 21.0% anatomical failure, with the anterior compartment being most often involved (12.8%). Symptoms of prolapse were reported by 9.4% of women and 1.5% required reoperation for prolapse recurrence. Similarly, Duan et al.^14^ retrospectively analyzed a cohort of 104 patients 9 years after USL suspension and reported a 91% cure rate (no prolapse beyond the hymen, symptoms of prolapse, or reoperation/pessary use). The anterior compartment was most affected by POP recurrence beyond the hymen (6.7%), followed by the posterior compartment (2.9%).

The pattern of recurrence after USL suspension described in these papers were reproducible and consistent with our series. The anterior compartment was confirmed to be the most frequent site of recurrence, whereas apical relapse was unlikely to occur after USL suspension. Similar to our data, it was a general finding that most women with anatomic recurrences are not symptomatic, and few of them require reoperation. This might indicate that even when recurrence is associated with bulging symptoms, they may be mild and only minimally affect quality of life. Excellent PGI‐I scores in our cohort of patients seem to confirm this hypothesis.

As a consequence, according to our data and previous reports, it is reasonable to consider high USL suspension an effective procedure for anatomic restoration and symptom relief even in the long term. Ureteral injuries represent a feared complication of this procedure and may act as a deterrent to its popularization for clinical use. According to the meta‐analysis by Margulies et al.,^19^ this complication may occur in up to 11% of procedures. However, the mean rate was estimated to be 1.8%, which is consistent with our data. Moreover, diagnostic cystoscopy with contrast dye allows early identification and intraoperative management, thus reducing long‐term sequelae. Recently, intraoperative power Doppler ultrasound has been proposed as a non‐invasive method to evaluate ureteral patency during pelvic surgery.^20^ As most ureteral obstructions can be resolved with the removal of the offending uterosacral suspension suture(s), the need to perform ureteral implantation to recover from ureteral injury is estimated to be only 0.6%.^19^

Although a short‐term positive impact on urinary, bowel, and sexual function after native‐tissue repair with the USL is well established, data on long‐term functional outcomes are scarce.^1^ This is particularly relevant, considering that aging is directly related to the prevalence of pelvic floor disorders, such as urinary incontinence, overactive bladder, constipation, and sexual inactivity.^21^ Schiavi et al.^18^ demonstrated an improvement in POP‐related quality of life and sexual function at 8.9 years of follow up after USLs suspension according to P‐QoL and international consultation on incontinence questionnaire‐urinary incontinence short form questionnaires, and prolapse/urinary incontinence sexual questionnaire, female sexual function index (FSFI), and female sexual distress scale‐revised scores, respectively.^18^ Similarly, Silva et al.^16^ evaluated 5‐year functional outcomes of USL suspension for uterovaginal or vaginal vault prolapse repair, and recorded a persistent positive impact on bladder function according to postoperative incontinence impact questionnaire/ urogenital distress inventory scores, with a significant improvement in all domains (irritative, obstructive, and stress) compared with preoperatively. Moreover, in sexually active women, 94% were satisfied with their sexual activity after surgery, despite an abnormal FSFI desire score.^16^ In our series, we observed a persistent positive impact of urinary function, in terms of reduction of stress urinary incontinence and voiding symptoms. Moreover, despite the significant aging of patients due to the very long follow‐up time, no detrimental effects were observed for bowel function and sexual well‐being.

Several risk factors for POP recurrence have been previously proposed. However, most studies evaluated the effect in the short to medium term, whereas there is a paucity of data about the long‐term impact. We found a protective role of menopausal status, anterior repair, and posterior repair towards surgical failure. The role of additional surgical procedures in preventing recurrence is intuitive. Proper recognition of pelvic floor vaginal compartment defects and appropriateness of additional surgical procedures—such as anterior and/or posterior repair—clearly represents a crucial factor in the success of POP surgery. On the contrary, lack of identification and correction of all anatomic defects may expose women to a higher risk of recurrence. In our previous work, we demonstrated that anterior repair was a protective factor for anatomical recurrence of stage 2 or more associated with bulging symptoms (*P* = 0.002).^8^ Similarly, Chen et al.^22^ found that a suboptimal correction of anatomic defects—defined as the immediate postoperative finding of apical prolapse of at least stage—was a significant risk factor for surgical failure.^22^ In this series, despite the relatively high rates of anterior and posterior repair (respectively, 89.2% and 74.6%), the lack of additional procedure represented a significant risk factor for reoperation. This may indicate that a certain grade of pelvic floor damage is probably widespread in all vaginal compartments and intraoperative recognition somehow underestimates defects.

On the contrary, the role of premenopausal status as a risk factor for recurrence may at first seem contradictory, because menopause and increasing age are well‐established risk factors for prolapse development. However, prolapse development in young fertile women might indicate a greater grade of pelvic floor damage and/or a poorer connective tissue quality. This hypothesis is consistent with the observation of histologic alteration in the connective tissue of the vesico‐vaginal fascia of patients with POP recurrence compared with controls.^4^ Moreover, previous studies have reported a higher risk of POP recurrence in younger patients using different cut‐offs.^23^, ^24^, ^25^

To our knowledge, this is the first study analyzing 10‐year outcomes after native‐tissue repair for uterovaginal prolapse. Strengths of our study include the long follow up, homogeneous and large population, multimodal evaluation of surgical success, and analysis of risk factor impact. A limitation is the retrospective study design. Another limitation is the loss at follow up, which may also include patients with a recurrence who seek help at another institution, so underestimating the failure rate. However, our loss rate was consistent with those of previous similar works.

In conclusion, our study demonstrated that native tissue repair through high USL suspension is a safe and effective procedure for the treatment of uterovaginal prolapse, with long‐lasting effectiveness over time and persistence of functional benefits even 10 years after the index surgery. Premenopausal status and lack of anterior and posterior repair represented long‐term risk factors for recurrences.

## CONFLICTS OF INTEREST

The authors have no conflicts of interest.

## AUTHOR CONTRIBUTIONS

All authors contributed to project development, data collection, data analysis, and manuscript writing.

## Data Availability

No. Research data are not shared.
